# Colocolic Intussusception in an Adult Patient Secondary to Caecal Adenocarcinoma

**DOI:** 10.1155/2021/5534918

**Published:** 2021-04-21

**Authors:** Hicham Elmajdoubi, Marouane Baiss, Mohamed El Absi, El Hassan El Faricha El Alami, Mohamed El Ouanani, El Mahjoub Echarrab, Mohamed El Amraoui, Abdelkader Errougani

**Affiliations:** Surgical Emergency Department, Avicenna Hospital, Mohammed V University, Rabat, Morocco

## Abstract

Acute intestinal invagination is the pathology of infants and small children. Its occurrence in adults is rare, and it represents 1 to 5% of intestinal occlusions often leading to the discovery of an organic cause that may be tumor. We report the case of a 72-year-old patient admitted to the emergency room of Ibn Sina Rabat, Morocco, for intestinal occlusion. The abdominal CT scan showed a voluminous intestinal invagination on a very probable heterogeneous digestive mass. The treatment was an open right hemicolectomy. The histopathological examination of the surgical specimen concluded a colonic well-differentiated adenocarcinoma with a 30% mucinous component. By review of literature, we discuss diagnostic and therapeutic procedures in emergency.

## 1. Introduction

Intestinal invaginations are rare in adults, and they represent 1 to 5% of intestinal occlusions often leading to the discovery of an organic cause that may or may not be tumoral, unlike in children, where the majority of cases are primitive.

It is defined by the incorporation of an intestinal segment into the one immediately downstream.

Abdominal CT scan can diagnose easily the invagination, but the characterization on the underlying lesion is much more difficult, if not impossible.

Treatment is always surgical. We report the case of an elderly woman being treated in the emergency room of the IBN SINA Rabat hospital.

## 2. Case Report

A 72-year-old female with no significant past medical history presented with diffuse abdominal pain, vomiting, and subocclusive syndrome for 10 days without any notion of rectorrhagia, in a context of deterioration of the general state and apyrexia.

At the time of presentation to the emergency room, her temperature was 37 C, blood pressure was 110/60 mm Hg, pulse was 60 beats per minute, respiratory rate was 16 breaths per minute, and oxygen saturation was 99% while breathing ambient air.

**Figure 1:**
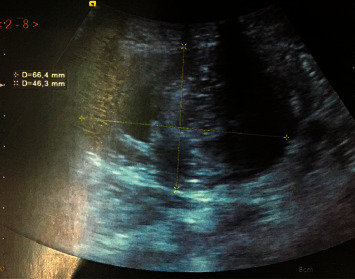
Abdominal ultrasound showing a left flank mass.

**Figure 2:**
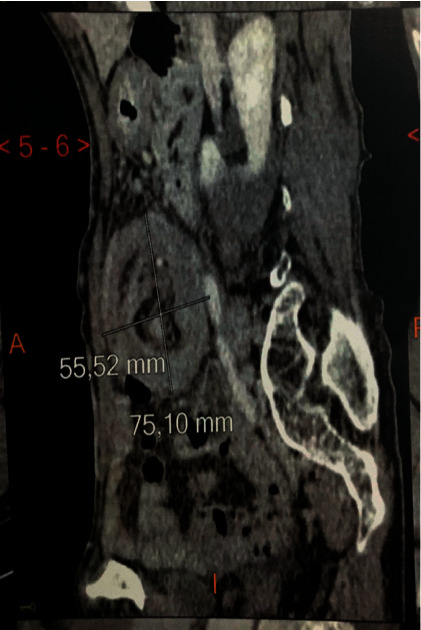
Abdominal CT scan showing large intestinal invagination (heterogeneous digestive mass).

**Figure 3:**
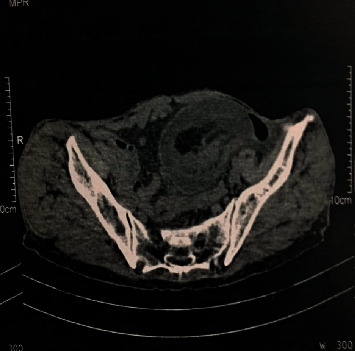
Abdominal CT scan showing large intestinal invagination (heterogeneous digestive mass).

The abdominal examination showed a slightly distended abdomen with the perception of a mass on the left flank extended to the left iliac fossa without adhesion to the abdominal wall. In addition, there was no hepatomegaly or splenomegaly; digital rectal examination does not revealed a palpable masse and rectum was empty; the rest of the clinical examination was without particularities; the biological assessment was normal; an abdominal X-ray radiography was performed showing some air fluid levels, Ultrasound revealed a mass on the left flank (Figure 1), and abdominal CT scan was performed for a better characterization, which showed a large intestinal invagination on a very probable heterogeneous digestive mass measuring 40 × 37 mm with invagination of the mesentery and signs of parietal pain on a digestive tumor mass (Figures 2 and 3).

**Figure 4:**
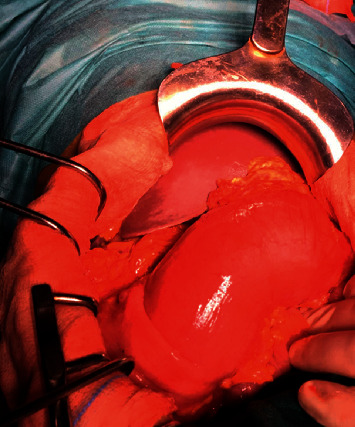
Operative image showing colocolic intussusception.

**Figure 5:**
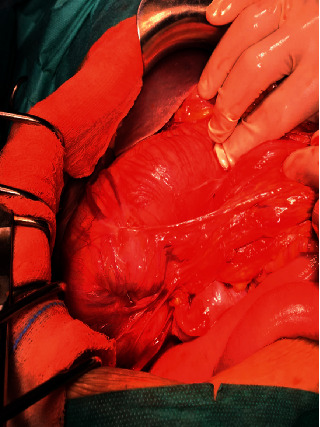
Operative image showing colocolic intussusception.

We performed an exploratory laparotomy that revealed colocolic intussusception caused by colonic tumor (Figure 4 and 5), an ascending 4.0 × 3.0 × 3.0 cm tumor as the lead point of intussusception into the proximal transverse colon with palpable mesentery lymph nodes Moreover, macroscopically, the tumor was ball-like with a regular surface and firm consistency. The cut surface was white. The appendix was aspirated by tumor; the exploration did not objectively reveal liver metastases or carcinoid nodules.

**Figure 6:**
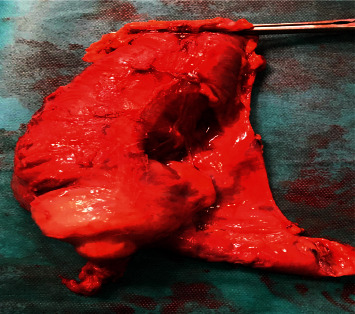
Right hemicolectomy for caecal mass.

The patient underwent a right hemicolectomy with terminolateral ileotransverse anastomosis in a single step (Figure 6). The postoperative course was uneventful, and she was discharged home after 6 days in good general condition.

Histopathological examination of the surgical specimen revealed a well-differentiated adenocarcinoma infiltrating the colonic musculosa with mucinous component estimated at 30%, tumor-classified PT2N0Mx.

## 3. Discussion

Intestinal invaginations are relatively rare in adults; they represent 2 to 4% of intestinal occlusions in adults [1], where an organic cause is found in 70 to 90% of cases and an idiopathic cause in 8 to 20%, whereas in children, intestinal invagination is primitive in 90% of cases [2, 3]. Adult intussusception of the small intestine is usually attributed to benign neoplasms, contrary to the large intestine, where malignancies constitute the majority [4].

The clinical presentation is polymorphic and often nonspecific: progressive installation of subocclusive syndrome over several days, acute intestinal occlusion, and nonspecific abdominal syndromes (altered transit, diffuse abdominal pain, and digestive bleeding), sometimes evolving over several months, with or without deterioration of the general state [5, 6].

Classically, in adults, the evolution is chronic with intermittent abdominal pain associated with subocclusive seizures. The acute form is mainly the prerogative of the ileoileal forms. For Mondor, the acute form would be the final stage of a chronic invagination for which an early diagnosis would not have been made [7]. This is the case of our patient who reported intermittent abdominal pain with a subocclusive syndrome before the installation of the acute picture.

The clinical presence of a palpable mass is a sign of great value in particular, if it appears to be of different location and consistency during repeated examinations it corresponds to the invagination coil, which should be carefully sought in the right and left lateral decubitus, in the dorsal decubitus, and Trendelenburg position [8].

Abdominal X-ray shows air fluid levels related to the occlusive syndrome, which is the case of our patient. Abdominal ultrasound can be useful to visualize the classical target sign with a thick peripheral hypoechoic band with central echogenicity zone. These correspond to the bowel wall surrounding hyperecohoic mesenteric fat contained within the intussusception, and it often remains hindered by the presence of air.

Abdominal CT scan confirms the diagnosis of the occlusive syndrome and shows the invagination, its precise location, and etiology. It can detect an organic cause in 71% of cases [9]. In CT, the typical target-like image consists of a thickened hyperdense external segment circumscribing a hypo- or hyperdense eccentric ring depending on the underlying cause and a stratified tissue ring with hypodense or discreetly hyperdense serous edema corresponding to the oedematous walls of the invaginated loop [10].

Treatment modalities for intussusception are variable. Because ileocolic is the most common type seen in children, reduction is often successful by using pneumatic or hydrostatic edema. Moreover, enteroenteric intussusceptions usually reduce spontaneously. This approach should be employed with great caution to avoid missing potentially serious underlying conditions such as malignancy. The operative approach is necessary in patients who present with bowel obstruction, those with mass seen on imaging, those with constitutional symptoms of malignancy (such as weight loss, anorexia and night sweats), and those with colocolic and ileocolic intussusception (given their higher association with malignancy) [11].

When surgical intervention is performed, if underlying malignancy is suspected, oncologic en bloc resection of the involved intestine and the associated mesentery should be sought [12]. This is recommended to avoid perforations and possible dissemination of cancerous cells.

Laparoscopy is currently a real means of diagnosis and sometimes treatment of intestinal intussusception [13]. In the case of intestinal obstruction, it requires expertise in laparoscopic surgery due to the distension of the small intestines, which hinders vision and makes it difficult to mobilise them, with a high risk of iatrogenic wounds. The anastomosis is realized immediately either by mechanical sutures or manual anastomosis after right hemicolectomy for carcinoma.

The prognosis is related to the duration of evolution, the extent of the lesions, and the nature of the cause [14].

## 4. Conclusion

Colocolic invagination in adults remains a rare condition. The abdominal CT scan is essential for the diagnosis of the invagination and its etiology. Concerning the treatment, a carcinological resection is necessary when a tumor with evidence of malignancy is discovered.
